# The impact of the origin of surgical sperm retrieval on placental and embryonic development: The Rotterdam Periconception cohort

**DOI:** 10.1111/andr.12943

**Published:** 2020-11-29

**Authors:** Jeffrey Hoek, Willem P. A. Boellaard, Eva S. van Marion, Sten P. Willemsen, Esther.B. Baart, Régine P. M. Steegers‐Theunissen, Sam Schoenmakers

**Affiliations:** ^1^ Department of Obstetrics and Gynecology Erasmus MC University Medical Center Rotterdam Rotterdam The Netherlands; ^2^ Department of Urology Erasmus MC University Medical Centre Rotterdam Rotterdam The Netherlands; ^3^ Department of Biostatistics Erasmus MC University Medical Centre Rotterdam Rotterdam The Netherlands

**Keywords:** fertilization in vitro, placenta, spermatozoa, testicular sperm, trophoblast

## Abstract

**Background:**

In patients with azoospermia, pregnancy can be achieved after surgical techniques using sperm retrieved from the testis or epididymis, which can impact on DNA integrity and epigenetics. DNA of the fetus and placenta is equally derived from both parents; however, genes important for placental development are expressed from the paternal alleles. Therefore, the origin of sperm may affect fetal and placental development.

**Objectives:**

To investigate whether first‐trimester trajectories of embryonic and placental development of pregnancies conceived after intracytoplasmic sperm injection (ICSI) with testicular sperm extraction (TESE) or microsurgical epididymal sperm aspiration (MESA), are different from pregnancies after ICSI with ejaculated sperm or natural conceptions.

**Materials and methods:**

A total of 147 singleton ICSI pregnancies, including pregnancies conceived after TESE (n = 23), MESA (n = 25) and ejaculated sperm (n = 99), and 380 naturally conceived and 140 after IVF treatment without ICSI were selected from the prospective Rotterdam periconception cohort. Crown‐rump length (CRL), embryonic volume (EV), Carnegie stages, and placental volume (PV) at 7, 9, and 11 weeks of gestation were measured using 3D ultrasound and virtual reality technology.

**Results:**

Linear mixed model analysis showed no differences in trajectories of CRL, EV, and Carnegie stages between pregnancies conceived after ICSI with testicular, epididymal, and ejaculated sperm. A significantly positive association was demonstrated for PV between pregnancies conceived after TESE‐ICSI (adjusted beta: 0.28(95%CI: 0.05‐0.50)) versus ICSI with ejaculated sperm. Retransformation to original values showed that the PV of pregnancies after TESE‐ICSI is 14.6% (95%CI: 1.4%‐25.5%) larger at 11 weeks of gestation compared to ICSI pregnancies conceived with ejaculated sperm.

**Discussion and Conclusion:**

Here we demonstrate that the first‐trimester growth trajectory of the placenta is increased in pregnancies conceived after TESE‐ICSI compared to those conceived after ICSI with ejaculated sperm. Findings are discussed in the light of known differences in sperm DNA integrity, epigenetics, and placental gene expression.

## 1 INTRODUCTION

Male factor subfertility is an increasing problem due to aging, obesity, and poor lifestyle, which can often be treated with lifestyle interventions.^1^, ^2^ In severe male factor subfertility, however, intracytoplasmic sperm injection (ICSI) is often successfully used to achieve pregnancy. Sperm used for assisted reproductive treatment (ART) can have different origins dependent on the underlying cause or diagnosis of the male factor. In cases of non‐obstructive azoospermia (NOA) sperm can be retrieved surgically by testicular sperm extraction (TESE). TESE can also be performed in the case of post‐vasectomy, iatrogenic, congenital or post‐infectious obstructive azoospermia (OA), or when vasovasostomy or microsurgical epididymal sperm aspiration (MESA) failed.^3^ In both cases the sperm is not (TESE) or only partially (MESA) transported through the epididymis. It is becoming increasingly clear that this transport through the epididymis is a key factor in sperm maturation and functioning.^4^ The epididymis secretes epididymal specific proteins, hormones, small non‐coding RNAs and these factors combined can influence sperm epigenetics, gene expression and modify the sperm surface.^5^ Although testicular sperm has not entered the anatomical part of the epididymis, it has the ability to activate the oocyte after ICSI. However, testicular sperm morphological quality is usually low and several studies indicate that the incidence of chromosomal abnormalities is increased in testicular sperm.^6^, ^7^ These abnormalities are associated with a decreased chance of implantation and lower ongoing pregnancy rates, possibly due to an increased aneuploidy rate in embryos.^7^, ^8^ However, a recent study in more than 340.000 IVF/ICSI cycles showed no clinical differences between pregnancies conceived with testicular, epididymal or ejaculated sperm regarding pregnancy rate and full‐term delivery.^9^ To overcome issues of timing and multiple surgical procedures for men, sperm can also be cryopreserved and thawed for later use. Several studies show similar treatment outcomes between fresh and frozen sperm.^10^, ^11^

The period of embryonic and placental growth in the first trimester of pregnancy is characterized by rapid cell multiplication and therefore vulnerable for alterations in both maternal‐ and paternal‐originated DNA integrity and epigenetic (re)programming. Interestingly, paternally imprinted genes are predominantly expressed in the placenta.^12^

Embryonic and placental growth can be reliably measured using three‐dimensional ultrasound (3D‐US) techniques in combination with virtual reality technology.^13^ This combination allows in‐depth perception and assessment of growth by measuring crown‐rump length (CRL) and embryonic volume (EV). Furthermore, it is possible to make an accurate assessment of the Carnegie stage based on internal and external morphological characteristics, as a marker of embryonic development.^14^

Since there are a number of differences regarding chromosomal constitution and epigenetics between sperm retrieved after TESE and MESA as compared to ejaculated sperm combined with a large preferential paternal expression profile of the placental genome, we hypothesize that embryonic and placental growth and development might differ between these groups. The aim of this study is to investigate whether placental and embryonic growth trajectories of ICSI pregnancies conceived with TESE and MESA are different from pregnancies after ICSI with ejaculated sperm, IVF or naturally conceived.

## 2 MATERIALS AND METHODS

### 2.1 Ethical approval

This study was conducted according to the guidelines laid down in the Declaration of Helsinki and all procedures involving patients were approved by the Medical Ethical and Institutional Review Board of the Erasmus MC, University Medical Centre, Rotterdam, the Netherlands (MEC‐2004‐227). Written informed consent was obtained from all female and male participants at enrollment.

### 2.2 Study population

Participants selected for this study were enrolled in the Rotterdam Periconception Cohort (Predict study).^15^ This is an ongoing open prospective tertiary hospital‐based cohort embedded in the outpatient clinic of the Department of Obstetrics and Gynecology of the Erasmus MC, University Medical Center Rotterdam, the Netherlands. The design of the cohort study has previously been published.^15^ Women and their partners were eligible for inclusion if they were at least 18 years of age and had an ongoing intrauterine singleton pregnancy and were less than 10 + 0 weeks of gestational age (GA). Participants were recruited for inclusion from November 2010 onwards.

For the current analysis, we included pregnancies of women who conceived after ICSI in combination with testicular, epididymal, cryopreserved or ejaculated sperm. For a general reference group, we included pregnancies of women who conceived through IVF with ejaculated sperm and women who conceived naturally. GA was either based on the exact conception date for ICSI and the reported last menstrual period for naturally conceived pregnancies. We excluded pregnancies conceived after using donor semen or oocyte donation, and pregnancies complicated by congenital malformations and intrauterine fetal demise. Furthermore, we excluded pregnancies of women with an irregular menstrual cycle (menstrual cycle of less than 25 days or more than 31 days) or from which no first day of the last menstrual period was known, since in these pregnancies GA is based on CRL, which is our main outcome parameter.

### 2.3 Sperm retrieval

Men with non‐obstructive azoospermia (NOA) all underwent TESE. In general, an obstructive component was excluded on medical history, physical examination, reproductive hormones (luteinizing hormone (LH), follicle‐stimulating hormone (FSH), and testosterone) and scrotal ultrasound. Karyotyping and Y‐chromosomal microdeletions were determined in all of these patients. All men with a diagnosed obstructive azoospermia (OA) initially underwent MESA. When motile sperm were microscopically detected, sperm cryopreservation was performed. When no motile sperm was found, TESE was performed. The latter group was excluded for analysis to create a homogenous TESE‐ICSI group with only NOA patients.

TESE was performed under local anesthesia with a standard bilateral open surgical biopsy technique. A transverse two centimeter scrotal incision was made, the tunica albuginea was incised for one centimeter (cm) and a small fragment (approximately 1.5 cm^3^) of testicular tissue was dissected using sterile glass slides. The collected tissue was subsequently minced and the resulting spermatogenic cell suspension was washed with HEPES‐HTF, followed by centrifugation at 900 g (Thermo Scientific Centrifuge) for 10 minutes. The pellet was resuspended in one ml HEPES‐HTF and subsequently diluted 1:1 with cryoprotectant (Test Yolk Buffer, Irvine Scientific, United States of America). When in 1 microliter suspension in 250 fields of view (FOV) at least one normal spermatozoa was found, cryopreservation was performed in straws (Cryo Bio System™, Irvine Scientific, United States of America) by placing them in liquid nitrogen vapor.

MESA was performed under local anesthesia with a scrotal incision and microsurgically opening of one or more tubuli in the caput epididymii. Spermatozoa were aspirated with a micropipette attached to a tuberculin syringe filled with HEPES‐buffered medium. The retrieved sperm was immediately diluted in HEPES‐buffered medium and cryoprotectant was added before cryopreservation.

For logistic reasons, our clinic always freezes TESE and MESA sperm. In men requesting semen cryopreservation because of upcoming spermato‐toxic treatments like chemotherapy for malignant diseases, fresh ejaculated sperm was diluted 1:1 with cryoprotectant (Test Yolk Buffer) and cryopreservation was performed in straws (Cryo Bio System™) by placing them in liquid nitrogen vapor. Semen cryopreservation was always performed prior to spermato‐toxic treatment.

### 2.4 Ovarian stimulation and embryo culture

Ovarian stimulation was performed by either a GnRH agonist or –antagonist followed by recombinant follicle‐stimulating hormone (FSH). Human chorionic gonadotrophin or a GnRH agonist were used as a trigger for final maturation of the oocyte and ovulation. Testicular, epididymal, and cryopreserved sperm were thawed and the sperm cells with best morphological and motile characteristics were selected for oocyte injection. After ovum pickup, the collected oocytes were injected with either frozen‐thawed testicular, epididymal or cryopreserved sperm or fresh ejaculated sperm. For the IVF treatment, fresh ejaculated sperm was washed and added to the dishes with oocytes.

All inseminated oocytes were cultured in G‐1 PLUS cleavage stage medium ((Vitrolife, Goteborg, Sweden) between January 2010 and November 2014 and SAGE 1‐StepTM medium (Origo/Cooper Surgical) from December 2014 onwards. All inseminated oocytes were cultured at 36.8 degrees Celsius with 7% oxygen and 5% carbon dioxide. The transfer of fresh embryos was performed on day 3 and the supernumerary embryos were cultured until day 4 and then cryopreserved. If a cryopreserved embryo was transferred, embryos were thawed and cultured overnight and transferred the following day corresponding to day 5 of embryo development.

### 2.5 Study parameters

Baseline characteristics and obstetric history were retrieved through self‐administered questionnaires covering details on age, ethnicity, and educational level. All data were verified at study entry by a researcher. Anthropometrics were measured by a researcher at study entry. Smoking and alcohol were defined as any consumption during the periconception period. Details regarding subfertility diagnoses, method of sperm retrieval and whether IVF or ICSI was used, were retrieved from the electronic patient files.

### 2.6 Ultrasound data

From November 2010 onwards women underwent three transvaginal ultrasounds, in the 7th, 9th, and 11th week of gestation. Ultrasound scans were performed with a 6‐12 MHz transvaginal probe using GE Voluson E8 equipment and 4D View software (General Electrics Medical Systems, Zipf, Austria).

To optimally make use of the depth information present in 3D‐ultrasound data, images were transferred to our Barco I‐Space (a Cave Automatic Virtual Environment–like virtual reality system).^13^ In the Barco I‐space, an interactive virtual reality hologram, which allows depth perception, was created. All measurements were performed by trained research staff.

### 2.7 Outcome variables

Outcome variables were generated multiple times in the first trimester of pregnancy, which gives the opportunity to study growth trajectories over time. CRL was measured three separate times per time point and embryo, and the mean of these measurements was used for analysis. EV measurements were performed once at each time point per embryo using a semi‐automatic method based on gray levels.^16^ Carnegie stages were determined once per time point to assess external morphological features of the embryo such as the development of the limbs and the curvature of the embryo, according to the protocol which was published previously.^14^ Placental volume (PV) in first trimester, also known as trophoblast volume, was measured once per time point per pregnancy using Virtual Organ Computer‐aided AnaLysis (VOCAL) (TM; GE Medical Systems, Zipf, Austria).^17^ In short, twelve sections of the placenta were obtained using a rotational step of 15º. Trophoblast and myometrium can be distinguished by their difference in echogenicity, thereby calculating the total pregnancy volume. The volume of the placenta can be calculated by subtracting the gestational sac volume from the total pregnancy volume.

### 2.8 Statistical analysis

To take the correlation between measurements of the same pregnancy into account, we used a linear mixed model. In the first trimester of pregnancy, we used this linear mixed model to assess the associations between the different origins of sperm and CRL, EV, Carnegie stage, and PV.

Furthermore, we used naturally conceived pregnancies and pregnancies after IVF with ejaculated sperm as a reference group for the different origins of sperm for ICSI.

For analysis, we used a square root transformation for the CRL measurements and a third root transformation for the EV and PV measurements, which led to linearity with GA. Carnegie stages were not transformed and used as continuous variable. For the graphs showing the EV and PV trajectories, the modelled EV and PV were retransformed to the original values.

In the first model, we adjusted for GA only. In the second model, we additionally adjusted for the paternal covariates age, smoking and alcohol use and maternal parity selected based on the characteristics of the study groups and literature.

*p*‐values <0.05 were considered statistically significant. All analyses were performed using SPSS package 24.0 (IBM SPSS Statistics, Armonk, NY).

## 3 RESULTS

### 3.1 Baseline

**Figure 1:**
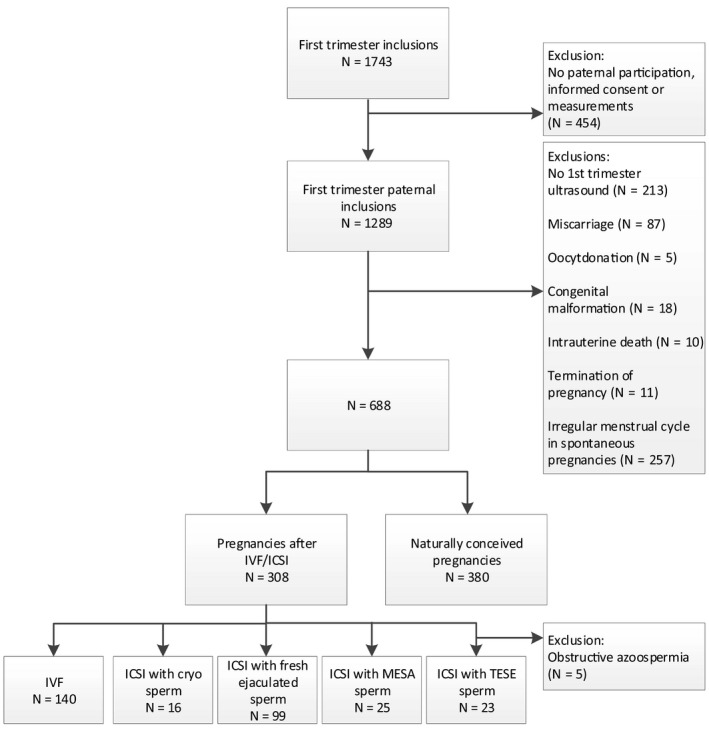
Flowchart of the in‐ and exclusions of the study population. ICSI, intracytoplasmic sperm injection; IVF, in vitro fertilization; MESA, microsurgical epididymal sperm aspiration; TESE, testicular sperm extraction

From a total of 1,743 pregnant women included in the cohort, 1,289 participated together with their male partner. Due to missing ultrasound data and women who did not undergo a first‐trimester ultrasound, 213 pregnancies were excluded. Furthermore, we excluded pregnancies because of miscarriage (n = 87), oocyte donation (n = 5), congenital malformations (n = 18), intrauterine fetal death (n = 10), and termination of pregnancy (n = 11) and cases with an irregular menstrual cycle prior to natural conception (n = 257). The remaining 688 included pregnancies comprised of 380 naturally conceived pregnancies, 140 pregnancies after IVF, and 168 pregnancies after ICSI. Of the included ICSI pregnancies, 28 were conceived after ICSI with TESE sperm. Five patients were excluded because of an obstructive azoospermia. The included 23 patients all underwent TESE because of a non‐obstructive azoospermia. In the ICSI pregnancy group, 25 resulted after ICSI with MESA sperm, 16 with cryopreserved sperm and 99 with freshly ejaculated sperm (Figure 1). There are no significant differences regarding the distribution of the origin of the sperm between pregnancies complicated by congenital malformations and pregnancies without congenital malformations (*P* = .09) and between pregnancies complicated by intra uterine fetal demise and pregnancies without intra uterine fetal demise (*P* = .73) (data not shown).

At baseline, there were no significant differences regarding paternal age, BMI, geographical background, education, alcohol use, and smoking between the different ICSI groups. Paternal age was significantly lower in the naturally conceived pregnancy group compared to the IVF group (33.6 vs 36.2 year respectively (*P < .01*) (Table 1). Regarding maternal factors at baseline, we found no statistical differences for age, BMI, geographical origin, education, and alcohol use. There was a significant difference regarding parity, where the percentage nulliparous in the naturally conceived group was lower compared to all other groups (*P < .001*).

**Table 1:** Baseline characteristics of the study population of (sub)fertile couples

Conception mode	ICSI with TESE sperm N = 23	ICSI with MESA sperm N = 25	ICSI with cryo sperm N = 16	ICSI with freshly ejaculated sperm N = 99	*p‐*value Differences over the ICSI groups	IVF N = 140	naturally conceived N = 380	*p‐*value Differences between all the groups
**Men**								
Age, years median (IQR)	32.8 (29.9‐36.5)	36.3 (32.9‐41.8)	35.2 (30.6‐38.1)	35.2 (32.1‐38.6)	0.08	36.2 (32.6‐39.9)	33.6 (30.5‐37.3)	**<0.01^a^**
BMI (measured), kg/m^2^ median (IQR)	26.2 (22.4‐29.1)	27.3 (23.9‐29.9)	26.6 (22.8‐31.2)	26.5 (24.4‐28.4)	0.59	26.0 (24.0‐28.6)	25.6 (23.7‐28.0)	0.23
Geographical origin								
Western, n (%)	18 (78.3%)	19 (76.0%)	15 (93.8%)	86 (86.9%)	0.31	108 (77.1%)	317 (83.4%)	0.24
Non‐Western, n (%)	5 (21.7%)	6 (24.0%)	1 (6.3%)	13 (13.1%)		32 (22.9%)	63 (16.6%)	
Education								
High, n (%)	10 (45.5%)	9 (37.5%)	8 (50.0%)	40 (43.5%)	0.99	65 (50.0%)	179 (49.6%)	0.77
Intermediate, n(%)	9 (40.9%)	11 (45.8%)	6 (37.5%)	40 (43.5%)		53 (40.8%)	126 (34.9%)	
Low, n (%)	3 (13.6%)	4 (16.7%)	2 (12.5%)	12 (13.0%)		12 (9.2%)	56 (15.5%)	
Alcohol use, n (%)	17 (81.0%)	17 (70.8%)	11 (73.3%)	60 (69.0%)	0.75	94 (75.2%)	272 (76.6%)	0.73
Smoking, n (%)	7 (33.3%)	4 (18.2%)	5 (33.3%)	20 (23.0%)	0.56	34 (27.2%)	120 (33.8%)	0.26
**Women**								
Age, years median (IQR)	30 (28.1‐33.2)	31.1 (28.8‐35.7)	30.8 (24.5‐35.2)	32.5 (29.5‐35.0)	0.09	34.6 (31.5‐38.1)	31.7 (29.1.‐34.9)	**<0.01^g^**
BMI (measured), kg/m^2^ median (IQR)	24.4 (21.7‐30.6)	22.5 (21.2‐28.2)	25.6 (22.8‐28.8)	24.9 (21.5‐28.0)	0.43	24.2 (21.7‐26.6)	24.2 (22.2‐28.2)	0.39
Parity, nulliparous, n (%)	18 (81.8%)	17 (68.0%)	12 (75.0%)	70 (70.7%)	0.7	94 (67.6%)	150 (39.5%)	**<0.001^f^**
Geographical origin								
Western, n (%)	16 (69.6%)	22 (88.0%)	15 (93.8%)	88 (88.9%)	0.08	113 (80.7%)	333 (87.6%)	0.11
Non‐Western, n (%)	7 (30.4%)	3 (12.0%)	1 (6.3%)	11 (11.1%)		27 (19.3%)	47 (12.4%)	
Education								
High, n (%)	7 (33.3%)	13 (54.2%)	5 (31.1%)	55 (57.9%)	0.12	84 (61.3%)	240 (63.3%)	0.06
Intermediate, n (%)	12 (57.1%)	9 (37.5%)	11 (68.8%)	33 (34.7%)		45 (32.8%)	117 (30.9%)	
Low, n (%)	2 (9.5%)	2 (8.3%)	0 (0.0%)	7 (7.4%)		8 (5.8%)	22 (5.8%)	
Alcohol use, n (%)	6 (30.0%)	6 (24.0%)	5 (33.3%)	19 (19.8%)	0.57	30 (22.1%)	149 (39.6%)	0.06
Smoking, n (%)	7 (35.0%)	2 (8.0%)	5 (33.3%)	8 (8.3%)	**0.002** ^b,c,d,e^	14 (10.2%)	60 (16.0%)	0.07

Note

Bold indicates significant results.

Posthoc test significant differences between: ^a^naturally conceived and IVF, ^b^TESE‐ICSI and fresh ejaculated sperm, ^c^TESE‐ICSI and MESA‐ICSI, ^d^cryo sperm‐ICSI and MESA‐ICSI, ^e^cryo sperm‐ICSI and fresh ejaculated sperm, ^f^naturally conceived and all other growth, ^g^IVF and all other growth.

Abbreviations: BMI, body mass index; ICSI, intracytoplasmic sperm injection; IQR, interquartile range; IVF, in vitro fertilization; MESA, microsurgical epididymal sperm aspiration; TESE, testicular sperm extraction.

Of the included pregnancies resulting after TESE‐procedures, the indication for ICSI treatment was because of either male (82.6%) or combined male–female factor subfertility (17.4%), with 100% being a non‐obstructive azoospermia (Table S1).

**Figure 2:**
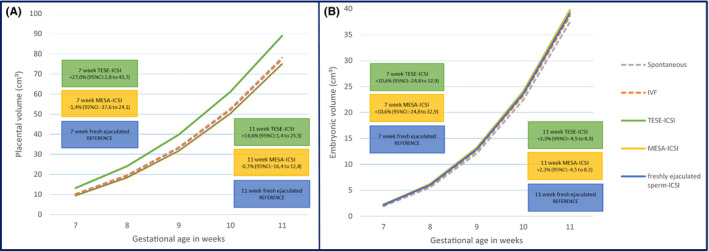
Growth trajectories for the pregnancies conceived after ICSI (TESE, MESA, ejaculated), naturally conceived pregnancies and IVF pregnancies regarding (A). placental volume and (B). embryonic volume. CI, confidence interval; ICSI, intracytoplasmic sperm injection; IVF, in vitro fertilization; MESA, microsurgical epididymal sperm aspiration; TESE, testicular sperm extraction

No differences were seen regarding the embryonic growth parameters CRL and EV when comparing the TESE and MESA‐ICSI groups with freshly ejaculated sperm‐ICSI (Table 2 and Figure 2B). Also, when comparing TESE‐ICSI with naturally conceived pregnancies, EV trajectories were not different (Model 2: TESE‐ICSI beta: 0.06 (95% confidence interval (CI): −0.01 to 0.13)) (Table 3 a). A significantly positive association was found for Model 1 regarding embryonic development estimated in Carnegie stages when comparing MESA‐ICSI with ICSI with freshly ejaculated sperm (beta: 0.38 (95%CI: 0.02 to 0.74)); however, this effect attenuated after correction for confounders (beta: 0.17 (95%CI: −0.18 to 0.52)) (Table 2).

**Table 2:** Betas of embryonic development (CRL, EV, Carnegie stages) and placental growth trajectories (PV) of different origins of sperm retrieval compared to ICSI with ejaculated sperm

	Model 1								Model 2							
	CRL		EV		Carnegie		PV		CRL		EV		Carnegie		PV	
	Beta (95% CI)	*p*‐value	Beta (95% CI)	*p*‐value	Beta (95% CI)	*p*‐value	Beta (95% CI)	*p*‐value	Beta (95% CI)	*p*‐value	Beta (95% CI)	*p*‐value	Beta (95% CI)	*p*‐value	Beta (95% CI)	*p*‐value
ICSI with ejaculated sperm	Reference		Reference		Reference		Reference		Reference		Reference		Reference		Reference	
ICSI with TESE sperm	0.02 (−0.08 to 0.11)	0.70	0.02 (−0.03 to 0.06)	0.54	0.14 (−0.25 to 0.52)	0.49	**0.23 (0.02 to 0.44)**	**0.03**	0,01 (−0.08 to 0.11)	0.80	0.02 (−0.03 to 0.07)	0.40	0.20 (−0.16 to 0.57)	0.28	**0.28 (0.05 to 0.50)**	**0.02**
ICSI with MESA sperm	0.01 (−0.08 to 0.09)	0.83	0.02 (−0.03 to 0.06)	0.51	**0.38 (0.02 to 0.74)**	**0.04**	‐0.01 (−0.21 to 0.20)	0.93	‐0.02 (−0.11 to 0.08)	0.73	‐0.003 (−0.05 to 0.05)	0.91	0.17 (−0.18 to 0.52)	0.34	0.03 (−0.20 to 0.27)	0.77
ICSI with cryo sperm	‐0.001 (−0.11 to 0.11)	0.98	‐0.002 (−0.06 to 0.06)	0.95	0.21 (−0.25 to 0.67)	0.37	0.01 (−0.27 to 0.29)	0.95	‐0.02 (−0.14 to 0.09)	0.71	‐0.01 (−0.07 to 0.05)	0.72	0.19 (−0.24 to 0.63)	0.38	0.06 (−0.23 to 0.35)	0.67

Note

Bold indicates significant results.

Model 1: Adjusted for gestational age. Model 2: Model 1 + adjusted for paternal age, smoking and alcohol and maternal parity.

Abbreviations: CI, confidence interval; CRL, crown‐rump length; EV, embryonic volume; ICSI, intracytoplasmic sperm injection; MESA, microsurgical epididymal sperm aspiration; PV, placental volume; TESE, testicular sperm extraction.

**Table 3:** Betas of embryonic development (CRL, EV, Carnegie stages) and placental growth trajectories (PV) of different origins of sperm retrieval compared to a. naturally conceived pregnancies and b. all cryopreserved sperm compared to freshly ejaculated sperm

	Model 1								Model 2							
	CRL		EV		Carnegie		PV		CRL		EV		Carnegie		PV	
	Beta (95% CI)	*p*‐value	Beta (95% CI)	*p*‐value	Beta (95% CI)	*p*‐value	Beta (95% CI)	*p*‐value	Beta (95% CI)	*p*‐value	Beta (95% CI)	*p*‐value	Beta (95% CI)	*p*‐value	Beta (95% CI)	*p*‐value
(A)																
Naturally	Reference		Reference		Reference		Reference		Reference		Reference		Reference		Reference	
IVF	**0.06 (0.003 to 0.12)**	**0.04**	**0.04 (0.01 to 0.06)**	**0.02**	**0.21 (0.01 to 0.41)**	**0.04**	‐0.01 (−0.11 to 0.08)	0.77	0.03 (−0.03 to 0.10)	0.32	0.03 (−0.01 to 0.06)	0.11	0.15 (−0.06 to 0.36)	0.16	0.03 (−0.07 to 0.14)	0.52
ICSI with ejaculated sperm	**0.08 (0.01 to 0.14)**	**0.02**	**0.05 (0.02 to 0.08)**	**0.003**	**0.27 (0.05 to 0.50)**	**0.02**	‐0.06 (−0.17 to 0.06)	0.34	0.06 (−0.01 to 0.13)	0.10	**0.04 (0.01 to 0.08)**	**0.02**	0.19 (−0.05 to 0.42)	0.12	‐0.07 (−0.19 to 0.06)	0.28
ICSI with TESE sperm	0.09 (−0.03 to 0.22)	0.13	**0.07 (0.01 to 0.13)**	**0.04**	0.41 (−0.01 to 0.84)	0.06	0.19 (−0.01 to 0.39)	0.06	0.09 (−0.04 to 0.22)	0.17	0.06 (−0.01 to 0.13)	0.11	0.27 (−0.16 to 0.71)	0.22	0.21 (−0.002 to 0.42)	0.05
ICSI with MESA sperm	0.08 (−0.04 to 0.20)	0.17	**0.06 (0.004 to 0.12)**	**0.04**	**0.65 (0.25 to 1.04)**	**0.001**	‐0.06 (−0.26 to 0.13)	0.53	0.01 (−0.11 to 0.14)	0.83	0.03 (−0.03 to 0.10)	0.34	0.26 (−0.16 to 0.68)	0.22	‐0.04 (−0.27 to 0.18)	0.71
ICSI with cryo sperm	0.08 (−0.07 to 0.23)	0.29	0.05 (−0.02 to 0.12)	0.18	0.49 (−0.03 to 1.00)	0.07	‐0.02 (−0.29 to 0.24)	0.86	0.08 (−0.08 to 0.23)	0.33	0.05 (−0.03 to 0.13)	0.20	0.46 (−0.07 to 0.98)	0.09	‐0.01 (−0.27 to 0.26)	0.97
(B)																
ICSI with ejaculated sperm	Reference		Reference		Reference		Reference		Reference		Reference		Reference		Reference	
ICSI with cryo sperm (TESE, MESA and cryo)	0.01 (−0.05 to 0.07)	0.75	0.01 (−0.02 to 0.04)	0.50	0.26 (−0.01 to 0.52)	0.06	0.09 (−0.06 to 0.24)	0.26	‐0.01 (−0.07 to 0.06)	0.84	0.004 (−0.03 to 0.04)	0.82	0.19 (−0.06 to 0.43)	0.14	0.14 (−0.02 to 0.31)	0.09

Note

Bold indicates significant results.

Model 1: Adjusted for gestational age. Model 2: Model 1 + adjusted for paternal age, smoking and alcohol and maternal parity.

Abbreviations: CI, confidence interval; CRL, crown‐rump length; EV, embryonic volume; ICSI, intracytoplasmic sperm injection; IVF, in vitro fertilization; MESA, microsurgical epididymal sperm aspiration; PV, placental volume; TESE, testicular sperm extraction.

Model 1 showed a significantly positive association (beta: 0.23 (95% confidence interval (CI): 0.02 to 0.44)) regarding PV in the TESE‐ICSI group when compared to ICSI with freshly ejaculated sperm (Table 2). After adjustment for paternal covariates age, smoking, alcohol, and maternal parity (Model 2) the significantly positive association remains (beta: 0.28 (95%CI: 0.05 to 0.50)). Retransformation of the betas to the original values showed that PVs of pregnancies after TESE‐ICSI are 14.6% (95%CI: 1.4% to 25.5%) larger at 11 weeks of gestation as compared to pregnancies after fresh ejaculated‐ICSI (Figure 2A).

To investigate the effect of cryopreservation, we pooled the groups with TESE and MESA treatment and cryopreserved sperm, and compared them with the group of ICSI with freshly ejaculated sperm. Again, we found no statistically significant differences regarding trajectories of CRL, EV, PV, and Carnegie stage (Table 3 b).

## 4 DISCUSSION

In this study, we show that pregnancies conceived after TESE‐ICSI compared with ejaculated sperm‐ICSI are associated with trajectories of increased PV in the first trimester of pregnancy, with an estimate of 14.6% increase at 11 weeks GA. Furthermore, we found no differences between TESE‐ICSI and ICSI with ejaculated sperm regarding embryonic growth and development as measured by CRL, EV and Carnegie stages.

The placenta is initiated with the formation of trophectoderm, which is part of the developing blastocyst. After fusion of the trophoblast cells, two placental lineages are formed, the syncytiotrophoblasts and the cytotrophoblasts. After invasion of the syncytiotrophoblasts into decidualised endometrium, the syncytiotrophoblast cells come in contact with the maternal blood supply. The role of paternally expressed (and thus maternally imprinted) genes on early placental growth and development is only partially established.^12^ Imprinted genes have a parent‐of‐origin effect by preferential expression of either the maternal or paternal inherited allele, emphasizing the essential influence of paternally expressed genes for early placental and embryonic development. Shortly after conception, a global loss of DNA methylation is initiated, followed by a remethylation starting from the blastocyst stage.^18^ However, since imprinted genes are largely unaffected by the demethylation wave, the epigenetic profile of the sperm cells at imprinted genes obtained during spermatogenesis is directly passed on to the resulting zygote and maintained in the (extra)embryonic tissues. Several paternally expressed genes, such as the *Paternally Expressed Gene* (*PEG*) *3* and *PEG 1*, all important in early growth and development, have shown to already be expressed in trophoblast cells.^19^, ^20^ Animal studies showed that a knockout model of *PEG 3* resulted in a significant reduction of placental size, whereas a knock out model of *PEG 1*, which is normally expressed in the invasive syncytiotrophoblasts, showed severe embryonic and placental growth restriction.^21^ Interestingly, previous research showed reduced DNA methylation in testicular sperm of azoospermic men of the imprinted gene *H19* compared to sperm of fertile men.^22^ The first trimester of pregnancy represents a period of rapid cell division and development, where differences in epigenetic regulation of genes involved in growth can have a large impact. So far, no studies are known investigating differences in epigenetic marks, such as DNA methylation, in neonates conceived after using TESE sperm. Considering our observation on the early developmental differences in placental volume in the first trimester, this would be of interest.

All testicular and epididydimal extracted sperm is cryopreserved after collection and thawed at the moment of oocyte pickup. Cryopreservation of sperm has been found to significantly alter sperm DNA methylation.^23^, ^24^ However, despite these reported epigenetic variations, we found no differences regarding embryonic and placental growth and development after pooling of all groups using cryopreserved sperm (TESE, MESA, and cryopreserved sperm) compared to ICSI with freshly ejaculated sperm. We cannot distinguish between the influence of the origin of the sperm or cryopreservation since in our clinic cryopreservation of the sperm is always used after TESE and MESA.

The finding of larger PV trajectories in early pregnancies conceived after TESE‐ICSI can also be explained by differences in DNA integrity due to DNA damage. Although the epidydimis is thought to play an important role in sperm maturation, a recent meta‐analysis showed that sperm DNA damage, measured by sperm DNA fragmentation, can be significantly higher in freshly ejaculated sperm compared to testicular sperm.^25^ The lower sperm DNA fragmentation can explain the larger placental development, in terms of better development with improved sperm DNA integrity. PV is associated with birth outcomes: pregnancies ending in miscarriage had smaller placental volumes during the first trimester as compared with those that result in a livebirth, indicating the potential beneficial role of using testicular sperm, since we found placental volume to be increased.^17^, ^26^ A confounding factor can be maternal smoking, since significantly more women smoked in the TESE‐ICSI group. However, since smoking is associated with smaller placental growth in the first trimester of pregnancy, PV is hypothesized to be even larger after using testicular sperm for ICSI in women who do not smoke.

Previous research investigating the association between surgically retrieved sperm and pregnancy outcomes comprised of far more participants than our study and mentioned no significant differences between testicular, epididymal, and ejaculated sperm regarding the birth outcomes birthweight and preterm birth.^27^, ^28^, ^29^ The aim of our study was to gain more insight into the early (patho)physiology of the role of the origin of sperm and embryonic and placental development, not to study pregnancy outcomes. Our study was not powered to detect differences regarding pregnancy outcomes. The posthoc sample size calculation using an α‐level of 0.05 and power of 80%, revealed that at least 80 participants in the total study group are needed to accurately show significance regarding placental volume, which were present in our study. This study revealed the magnitude of effect sizes which will aid to determine an optimal sample size for future larger studies or randomized controlled trials.

No effects were established regarding embryonic growth, as measured by CRL and EV, between the different sperm origins. Since paternally expressed genes are predominantly expressed in the placenta, we expected to show periconceptional paternal effects on PV trajectories. Our group previously showed that IVF‐ICSI pregnancies exhibit larger embryonic growth compared to naturally conceived pregnancies indicating that the procedure itself can induce differences regarding embryonic development.^30^ Another possible explanation for a lack of detectable effect of TESE sperm on CRL and EV, could be due to the strong impact of the IVF‐ICSI procedure itself on epigenetic reprogramming of the embryo and endometrial receptivity.^31^, ^32^

Our study has several strengths and limitations. A strength is the availability of multiple serial 3‐D ultrasounds in the first trimester of the same pregnancy and the possibility to precisely assess several morphogenic features of embryonic and placental growth and development. Our group previously showed that early PV can reliably be measured with very high intra‐class correlation coefficients (ICC > 0.95).^33^ In the present study, we confirm other studies that showed no significant differences regarding PV between naturally conceived pregnancies (n = 84) and IVF‐ICSI pregnancies (n = 70) (39.8cm^3^ and 40.2cm^3^ respectively).^34^, ^35^ Moreover, unique in our study is the comparison of differences in embryonic and placental growth trajectories in subgroups of ICSI with TESE, MESA and ejaculated sperm versus IVF (with ejaculated sperm) with naturally conceived pregnancies as reference groups. Furthermore, we were able to differentiate between the effects of the cryopreservation procedure and the direct effect of TESE sperm itself by showing results of all cryopreserved sperm compared to ICSI with freshly ejaculated sperm. A limitation of our study remains a relatively low number of patients in the different subgroups. Since this study is an observational cohort, we adjusted for potential confounders; however, residual confounding cannot be fully excluded. We used self‐administered questionnaires, which are verified face to face at the first consultation by a trained research nurse, to obtain information regarding baseline variables. Social desirable answers can be expected regarding smoking and alcohol use. However, because of an expected random effect on both patient groups suffering from subfertility, we assume that confounding of the effect estimates is limited. In epidemiology, observational studies are performed as a first step for hypothesis testing and showing associations. Therefore, the results should be interpreted with causation and causality should be shown in a randomized controlled design. Our study population was recruited from a tertiary hospital. The indication for cryopreservation of sperm, for example, includes hematological or testicular cancer, which limits extrapolating of our results to a general population. Despite homogenization of the groups per different surgically retrieval procedure (only cases with NOA in the TESE group, and OA in the MESA group), other differences between groups are present, such as the cause of infertility. Since our study population is too small to correct or stratify for these factors, future larger studies should incorporate these factors in sample size calculation and analyses. Furthermore, we did not investigate birth outcomes, which was not an outcome in this study and therefore not powered accordingly.

## 5 CONCLUSION

We show that placental growth trajectories in the first trimester of pregnancy are increased in pregnancies conceived after TESE‐ICSI as compared to ejaculated sperm. No significant differences are shown regarding embryonic development measured as CRL, EV and Carnegie stages. These findings might be partially explained by differences in DNA damage, chromosomal constitution and epigenetics of testicular sperm as compared to ejaculated sperm. After this explorative study, future research should validate these findings in larger cohorts, including investigating possible associations with birth outcomes. Exploring the underlying pathofysiological mechanisms by measuring sperm DNA damage, placental and also the neonatal epigenome will provide insights that help optimize preconceptional health and counseling. This will help to further improve pregnancy chances and birth outcomes in subfertile couples with male factor subfertility.

## CONFLICT OF INTEREST

The authors report no conflicts of interest.

## AUTHOR'S CONTRIBUTIONS

RST and SS initiated the research question and supervised all aspects of the study. EB was responsible for the ICSI treatment in the laboratory. JH, WB and EvM contributed to data acquisition. SW and RST supervised the statistical procedures of the manuscript. JH, WB and SS wrote the first version of the manuscript. All authors contributed to the writing and the critical revisions of the manuscript and all authors approved and authorized the final version.

## Supporting information

Table S1
