# Supplementary material for: The impact of the origin of surgical sperm retrieval on placental and embryonic development: The Rotterdam Periconception cohort
Source: Andrology. 2020 Nov 29;9(2):599–609. doi: 10.1111/andr.12943 (PMC7986236; doi:10.1111/andr.12943)
Supplement: Supplementary file 1 — Table S1 [file ANDR-9-599-s001.docx]

**Supplemental table 1**: Diagnosis category per ART treatment

|  | **ICSI**  **TESE**  **Sperm**  **(n=23)** | **ICSI**  **MESA**  **Sperm**  **(n=25)** | **ICSI**  **ejaculated sperm**  **(n=99)** | **IVF**  **(n=140)** |
| --- | --- | --- | --- | --- |
| Female factor subfertility | 0 (0.0%) | 0 (0.0%) | 21 (21.2%) | 119 (85.0%) |
| Combined female-male subfertility | 4 (17.4%) | 3 (12.0%) | 17 (17.2%) | 10 (7.1%) |
| Male factor subfertility | 19 (82.6%) | 22 (88.0%) | 56 (56.6%) | 10 (7.1%) |
| Missing | 0 (0%) | 0 (0%) | 3 (3.0%) | 1 (0.7%) |
| **Female factor diagnosis**  **of both female only and combined male-female** | N=4 | N=3 | N=38 | N=129 |
| Tuba | 1 (25%) | 0 (0%) | 4 (10.5%) | 21 (16.3%) |
| Endometriosis | 0 (0%) | 0 (0%) | 5 (13.2%) | 22 (17.1%) |
| Unexplained | 0 (0%) | 0 (0%) | 14 (36.8%) | 64 (49.6%) |
| Anovulation | 3 (75%) | 3 (100.0%) | 15 (39.5%) | 22 (17.1%) |
| **Male factor diagnosis**  **of both male only and combined male-female** | N=23 | N=25 | N=73 | N=20 |
| oligozoospermia | 0 (0.0%) | 0 (0.0%) | 73 (100%) | 20 (100%) |
| Obstructive azoospermia | 0 (0.0%) | 25 (100 %) | 0 (0.0%) | 0 (0.0%) |
| Non-obstructive azoospermia | 23 (100%) | 0 (0.0%) | 0 (0.0%) | 0 (0.0%) |
| Of which: |  |  |  |  |
| Normal karyotype, no Y chromosome microdeletions | 22 (95.7%) | 19 (76.0%) | n/a | n/a |
| Karyotype 47, XXY | 1 (4.3%) | 0 (0%) | n/a | n/a |
| CFTR gene mutation | 0 (0%) | 6 (24.0%) | n/a | n/a |

Data are presented as number of subjects (%). Abbreviations: IQR; interquartile range, IVF; in vitro fertilization, ICSI; intracytoplasmic sperm injection, TESE; testicular sperm extraction, MESA; microsurgical epididymal sperm aspiration, CFTR; cystic fibrosis transmembrane conductance regulator, n/a; not applicable.
